# Population pharmacokinetic analysis of intravenous voriconazole in cancer patients

**DOI:** 10.1371/journal.pone.0318883

**Published:** 2025-03-27

**Authors:** Zunaira Akbar, Muhammad Usman, Muhammad Aamir, Zikria Saleem, Muhammad Rehan Khan, Abdulwahab Alamri, Mohammed Salem Alharbi, Gamal Eldin Mohamed Osman

**Affiliations:** 1 Department of Pharmacy, The University of Lahore, Lahore, Pakistan; 2 Riphah Institute of Pharmaceutical Sciences, Riphah International University, Lahore, Pakistan; 3 Institute of Pharmaceutical Sciences, University of Veterinary and Animal Sciences, Lahore, Pakistan; 4 Faculty of Pharmacy, Bahauddin Zakariya University, Multan, Pakistan; 5 Shaukat Khanum Memorial Cancer Hospital and Research Centre, Lahore, Pakistan; 6 Department of Pharmacology and Toxicology, College of Pharmacy, University of Hail, Hail, Saudi Arabia; 7 Medicine Department, College of Medicine, University of Hail, Hail, Saudi Arabia; 8 Pediatrics Department, Faculty of Medicine, University of Hail, Hail, Saudi Arabia; Shiraz University of Medical Sciences, IRAN, ISLAMIC REPUBLIC OF

## Abstract

**Purpose:**

The pharmacokinetics of voriconazole have been studied across various populations but data specific to the Pakistani cancer population has not yet been reported. The aim of present study was to explore and identify covariates that affect pharmacokinetics of intravenous voriconazole in Pakistani cancer population.

**Methods:**

The therapeutic drug monitoring data from January1^st^, 2023 to December 31^st^, 2023 of cancer patients receiving intravenous voriconazole for systemic fungal infections were taken from electronic medical record of the hospital. The data were used for the development of population pharmacokinetic model using NONMEM. Impact of various covariates such as age, weight, sex, liver function test, serum creatinine, creatinine clearance, type of cancer (primary diagnosis) and type of fungal infection were assessed through stepwise covariate modeling. Bootstrap analysis and goodness of fit plots were used to evaluate robustness and predictive performance of final model.

**Results:**

One compartment model best described the included data with first order elimination. The value of voriconazole clearance was 6.17 L/h with interindividual variability of 83.7% while volume of distribution was 55.9 L. The clearance of voriconazole was significantly influenced by renal status of patients. Creatinine clearance and primary diagnosis were significant covariates affecting clearance of voriconazole in covariate analysis.

**Conclusion:**

The findings suggest that this model can be used for dosage adjustment based on creatinine clearance and primary diagnosis as they impact significantly on voriconazole clearance in cancer patients. This approach is especially valuable in resource-limited settings like Pakistan, where individualized therapy can enhance the safety and efficacy of antifungal treatment, addressing the unique clinical and demographic challenges in vulnerable populations.

## Introduction

Voriconazole effectively targets a broad spectrum of clinically significant pathogens such as *Candida* and *Aspergillus*. It is recommended as first line drug by Infectious disease society of America (IDSA) for aspergillosis and an alternative agent for candidemia [1]. Voriconazole works by inhibiting fungal cytochrome P450 dependent 14α-sterol demethylase, an enzyme crucial for converting lanosterol to ergosterol, thereby disrupting the synthesis of the fungal cell membrane [2]. After oral administration in adults, voriconazole is rapidly absorbed, reaching peak plasma concentrations (Cmax) within 1 to 2 hours when taken on an empty stomach [3]. It has an estimated absolute bioavailability of 96%, facilitating change from intravenous to oral administration when clinically appropriate. Voriconazole is distributed extensively into tissues, with a steady-state volume of distribution (Vss) of around 4.6 liters per kilogram [4]. In pediatric patients, the oral bioavailability of voriconazole is approximately 44.6%, which is notably lower compared to about 96% in adults [5]. The drug primarily binds to albumin, accounting for 25.5% of the total plasma protein binding, and to a lesser extent, to α1-acid glycoprotein, which makes up 4.8% of the overall plasma protein binding. It undergoes extensive metabolism, with only 2% excreted unchanged while 98% is primarily metabolized through oxidative processes [6].

Voriconazole pharmacokinetics exhibit substantial interpatient and intrapatient variability, which can be attributed to several factors. These include variations in the cytochrome (CYP) 2C19 enzyme due to genetic polymorphism, patient-specific characteristics such as weight, age and liver function, as well as interactions with other drugs [7]. The prevalence of slow metabolic genotypes differs between countries. Asians have a higher proportion of slow metabolic genotypes compared to Caucasian or Black populations, indicating that Asian populations are at an increased risk of experiencing elevated drug concentrations [8]. Compared to normal metabolizers of voriconazole, intermediate or poor metabolizers exhibit higher serum levels of the drug. Due to the higher prevalence of poor metabolizers in Asian populations compared to White populations, there is an increased prevalence of hepatotoxicity observed among Asians [9]. Additionally, voriconazole displays non-linear pharmacokinetics due to its saturable metabolism and capacity-limited elimination process. These factors pose a difficulty for clinicians in optimizing voriconazole dosing regimens [10].

The plasma concentration of voriconazole is directly linked to both its efficacy and toxicity. Studies have shown that low plasma concentration (<1 µg/ml) of voriconazole is associated with poor clinical outcomes, while high plasma concentrations (>5.5µg/ml) can lead to adverse effects such as neurological issues, visual disturbances, and hepatotoxicity [11]. Population pharmacokinetic modeling is mostly used to establish pharmacokinetic parameters for a population and to identify the factors contributing to pharmacokinetic variability [12]. Various population pharmacokinetic studies have reported significant variation by covariates age, body weight, aspartate aminotransferase/alanine aminotransferase (AST/ALT) and concomitant drugs on voriconazole pharmacokinetics [10,13–16] but these factors have not been evaluated in Pakistani cancer population. Additionally, the burden of renal dysfunction, delayed healthcare access, and comorbidities such as malnutrition and infection prevalence in cancer patients may further contribute to pharmacokinetic variability. These factors, combined with limited therapeutic monitoring facilities in resource-constrained settings, underscore the need for population-specific models to optimize dosing and improve clinical outcomes. The aim of present study was to explore and identify covariates that affect pharmacokinetics of intravenous voriconazole in Pakistani cancer patients to bridge the gap in antifungal pharmacokinetics literature for underserved populations.

## Materials and methods

### Ethical approval

Ethical approval was taken from Institutional review board and ethical committee of Shaukat Khanum memorial cancer hospital and research centre (EX-05-09-23-06).

### Study design and study center

A one-year retrospective study was conducted by collecting TDM data of cancer patients who received voriconazole between 1^st^ January 2023 to 31^st^ December 2023 at Shaukat Khanam memorial cancer hospital and research center, Lahore, Pakistan. This hospital is a leading cancer care institution providing comprehensive diagnostic, treatment, and research services. With 195-bed tertiary care facility this hospital specializes in comprehensive cancer treatment, including surgical oncology, medical oncology, radiation oncology, and supportive care services.

### Study population

All cancer patients who were prescribed intravenous (I/V) voriconazole for systemic fungal infections either empirically for suspected fungal infections or targeted treatment for aspergillosis, febrile neutropenia, fungal pneumonia, candidiasis (fluconazole resistant) or any other fungal infection were included in this study.

### Inclusion and exclusion criteria

All admitted adult and pediatric cancer patients irrespective of gender, type of cancer and type of fungal infection who received intravenous voriconazole from 1^st^ January 2023 to 31^st^ December 2023 for systemic fungal infections were included. Patients were excluded if they had missing TDM data or if serum voriconazole levels were not measured on the appropriate day due to delayed sampling. Patients with multiple admissions were excluded, and only the first admission was considered. Out of 488 admitted cancer patients with systemic fungal infections, data of 112 patients who received voriconazole were retrieved from EMR. However, 88 patient’s data with complete information and accurately measured levels were included in the study.

### Therapeutic drug monitoring of voriconazole

The TDM of voriconazole and few other drugs with narrow therapeutic window, significant inter- and intra-patient variability, and potential for serious adverse effects or sub-therapeutic dosing is routinely performed as part of daily patient care to ensure optimal drug efficacy and safety. The infectious diseases physician enters the order in hospital information management system (HIMS); if not, the ID pharmacist intervenes. According to hospital approved protocol, following the initiation of therapy, trough levels are measured on the 5th day before the morning dose. These levels are evaluated to ensure an optimal concentration range of > 1 µg/mL to < 5.5 µg/mL is achieved. For invasive fungal infections, the usual loading dose of voriconazole is 6 mg/kg q12h for first 24 hours and maintenance dose is 4 mg/kg q12h thereafter subsequent trough levels are ordered based upon patients’ clinical response. Serum concentration of voriconazole was measured using homogeneous enzyme immunoassay on Siemens Atellica CH-930. The method is validated for accuracy, precision, and sensitivity, ensuring reliable quantification of drug levels.

### Data collection

A review of electronic medical records (EMR) of the patients was done from 1^st^ October 2023 to 31^st^ March 2024 to collect data after getting ethical approval dated 27-Sep-2023. The patient’s informed consent was not needed because of retrospective nature of data however, the identity of the patients was concealed by using a separate code for each patient instead of his/her name or medical registration number. Patient demographics including age, gender, weight (kg) and clinical characteristic of patients including type of cancer, type of fungal infection, liver function test i-e aspartate aminotransferase (AST), alanine aminotransferase (ALT), alkaline phosphatase (ALP), Renal function test including serum creatinine (Scr), creatinine clearance (CLCR), administered dose and measured serum levels were recorded. The demographics of the patients and sampling information is provided in Table 1.

**Table 1:** Patient demographics and sampling data.

Patient demographics	Pooled data
**Gender n (%)**	
Male	47 (53.4)
Female	41(46.6)
**Age group n (%)**	
Adult	47 (53.4)
Pediatric	41 (46.6)
**Type of cancer n (%)**	
Leukemia	50 (56.8)
Lymphoma	14 (15.9)
Sarcoma	9 (10.2)
Breast cancer	8 (9.1)
Myeloma	4 (4.5)
Glioma	3 (3.4)
**Type of fungal infection**	
Suspected fungal infection	48(54.5)
Aspergillosis	31(35.2)
Fungal pneumonia	9 (10.2)
Age (years)	35(3-67)
Weight (kg)	30.85 (5.3-95.6)
AST(mean)	55.1
ALT (mean)	43.8
ALP (mean)	209.4
Scr (mean)	0.63
**Sample data**	
No.of samples	88
Dose (mg)	56-400
Trough concentration (μg/mL)	0.10 to 21.0

The data values are in median (range) unless otherwise specified.

### Population pharmacokinetic analysis

The population pharmacokinetic (popPK) modeling process was initiated with the Non-Linear Mixed Effect Modeling (NONMEM) software, version 7.4.4 (Icon Clinical Research LLC, New York, USA) which is supported by the PsN toolkit. The development of Non-linear Mixed Effect Modelling (NONMEM®) software has tremendously enhanced the ability to analyze sparse/TDM data [17]. The Pirana interface was used for model management, execution, validation, and report generation [18]. Initial model was created without covariates. The data was analyzed by one-compartment model as well as two compartment model, selecting the most appropriate model based on the least objective function value (OFV) and visual inspection of goodness-of-fit plots. Individual differences was modeled with an exponential approach, while sampling error and analysis errors were assessed using additive, proportional, and combined error models [19].

### Covariate analysis

After developing the base model, the impact of various covariates on the pharmacokinetic (PK) parameters of voriconazole was examined using stepwise covariate modeling (SCM). In this method, continuous covariates such as age, body weight, AST, ALT, ALP, and Scr, along with categorical covariates like gender, type of cancer and type of fungal infection were individually added to the base model. The reduction in the Objective Function Value (OFV) was monitored, with level of significance (α =  0.05), meaning a decrease of 3.84 points in OFV following the inclusion of a specific covariate was deemed significant for its inclusion in the model. This process was continued until no further factors remained to be included in the full model. Next, the process of backward elimination was initiated, where each factor included in the model was removed individually using a stringent criteria for level of significance (α =  0.05). A factor’s influence was considered significant if its removal resulted in an increase in the Objective Function Value (OFV) by 6.63 points. Process was continued until no additional factors remained for elimination from the full model, resulting in the final model.

### Model evaluation

The final model was obtained by including all significant covariates. Its predictive performance was assessed through goodness-of-fit analysis, which involved visually inspecting scatterplots of observed concentration (DV) against individual predictions (IPRED), DV against population predictions (PRED), Conditional Weighted Residuals (CWRES) against PRED, and CWRES against time post-dose. To evaluate the robustness and stability of the final model, a bootstrap analysis was conducted using PsN, running the final model 1000 times with auto-generated datasets through repeated sampling with various patient combinations. The estimated PK parameters from the final model were then compared with the median bootstrap estimates and the 95% confidence interval derived from the 2.5^th^ and 97.5^th^ percentiles of the distribution.

## Results

### Patient demographics and sampling data

The population pharmacokinetic model was developed by using the trough concentrations of voriconazole. Among 88 patients, 47 (53.4%) were male patients and 41 (46.6%) were female patients. The median age of the patients was 38 years with a range of 3 to 90 years and median body weight was 30.8 kg ranging from 5.3 to 95.6 kg. Majority of cancer patients were of leukemia 50 (56.8%) followed by lymphoma 14 (15.9%), sarcoma 9 (10.2%), breast cancer 8 (9.1%), myeloma (4 (4.5%) and glioma 3 (3.4%). Majority of patients were prescribed voriconazole for suspected fungal infections 48 (54.5%). The administered dose of voriconazole ranged from 56 mg to 400 mg while trough concentrations ranged from 0.1 μg/mL to 21 μg/mL.

### Population pharmacokinetic modelling

A one-compartment model with first-order elimination (SUBROUTINE ADVAN1 TRANS2) was used for data description due to lesser value of OFV (304) as compared to two compartment model with OFV (325). The estimation method utilized for determining the PK parameters of voriconazole was first-order conditional estimation with interaction (FOCE-I), while interindividual variability (IIV) was modeled using an exponential random effect. The residual variability between predicted and observed concentrations of voriconazole, which accounted for sampling and analysis errors, was described with a proportional error model.

### Covariate analysis

In stepwise covariate modeling, CLCR and type of cancer were identified as significant covariates for CL, leading to a 24.63-point reduction in the OFV upon their inclusion in the base model. The starting OFV of base model was 308.2 which was reduced to 304.3 after the inclusion of CRCL on CL. There was a further drop of 20.7 points resulting in OFV as 283.6. No other covariates were proved significant in forward inclusion. The included covariates were also proved significant during backward elimination step and no covariate was removed in this step. The Equations 1–6 describes the effect of type of cancer on CL.


$$IF\left(DISEASE.EQ.1\right)CL=6.17;Mostcommon$$ (1)

$$IF\left(DISEASE.EQ.2\right)CL=6.17\times \left(1+0.0191\right)$$ (2)

$$IF\left(DISEASE.EQ.3\right)CL=6.17\times \left(1+0.185\right)$$ (3)

$$IF\left(DISEASE.EQ.4\right)CL=6.17\times \left(1-0.817\right)$$ (4)

$$IF\left(DISEASE.EQ.5\right)CL=6.17\times \left(1-0.0233\right)$$ (5)

$$IF\left(DISEASE.EQ.6\right)CL=6.17\times \left(1+0.0881\right)$$ (6)

where disease (1) is leukemia, disease (2) is lymphoma, disease (3) is sarcoma, disease (4) is breast cancer, disease (5) is myeloma and disease (6) is Glioma.

**Fig 1:**
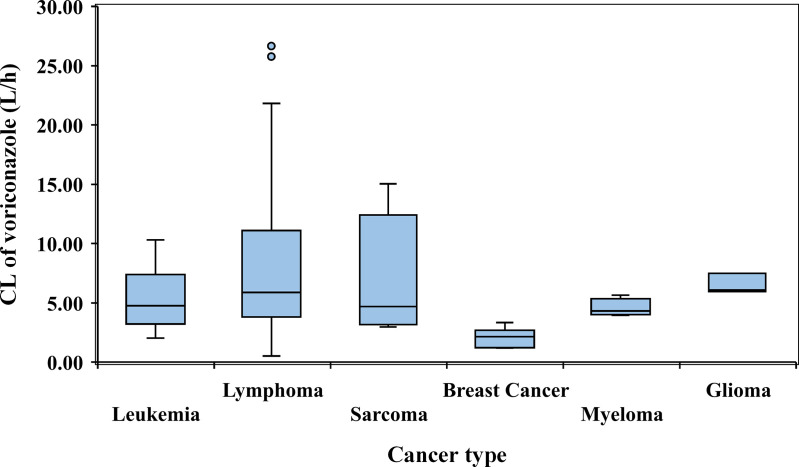
Box plot showing clearance of voriconazole in different cancer patients.

The effect of creatinine clearance on clearance of voriconazole can be described as


$${CL}_{j}=6.17*(1+0.0077*{\left({CRCL}_{j}-120\right)}^{\eta 1}$$ (7)

where Cl_j_ is the clearance of voriconazole in j^th^ individual and CRCL_j_ is the creatinine clearance of j^th^ individual. η1 is the interindividual variability in clearance and 6.17 is the median value of clearance in the population. Fig 1 represent comparison of voriconazole clearance in different cancer patients.

### Model evaluation

**Fig 2:**
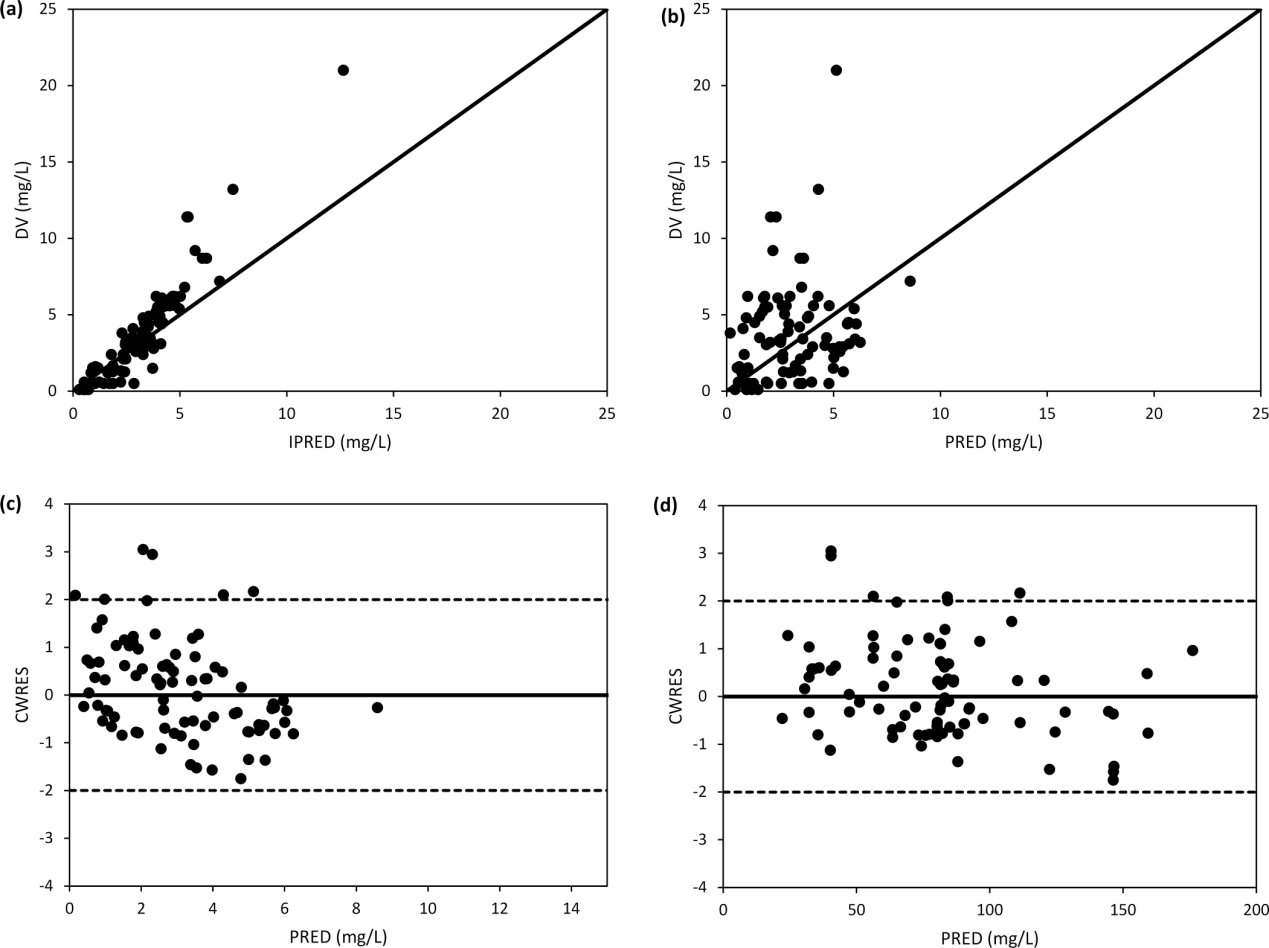
Goodness-of-fit plots of final model showing observed concentrations vs. individual predictions (a), observed concentrations vs. population predictions (b), CWRES vs. population predictions (c) and CWRES vs. time after dose (d).

The predictive accuracy of the final model was ascertained by goodness-of-fit plots as shown in Fig 2. The uniform distributions of observed concentrations versus individual predictions and population predictions around identity line are shown in Fig 2a and 2b respectively. In the scatterplots of conditional weighted residuals (CWRES) versus population predictions and time post-dose, CWRES were randomly distributed around the zero line, with over 95% of the values falling within the acceptable range of -2 to 2, as depicted in Fig 2c and 2d. The final model’s stability was verified by comparing bootstrap estimates with the final model’s parameter estimates. There was a close alignment between the final model’s parameter estimates and the median values of 1,000 bootstrap estimates, in conjunction with 95% confidence interval as shown in Table 2.

**Table 2:** Final model parameter estimates compared with bootstrap estimates.

Parameters	Final estimate	Bootstrap estimate	95% CI
OFV	283.6	277.5	235.3 to 312.80
CL(L/h)^a^	6.17	5.97	3.95 to 7.77
Vd (L)^b^	55.9	54.5	39.9 to 81.5
Proportional error	0.44	0.413	0.29 to 0.58
IIV-CL (%)	83.72	80.13	48.41 to 104.86
CRCL-CL^c^	0.0077	0.0074	0.0039 to 0.0088
CL-DISEASE 1^d^	0.0191	0.0095	-0.382 to 0.777
CL-DISEASE 3^e^	0.185	0.176	-0.403 to 2.01
CL-DISEASE 4^f^	-0.817	-0.807	-0.919 to -0.593
CL-DISEASE 5^g^	-0.0233	-0.0039	-0.365 to 0.553
CL-DISEASE 6^h^	0.0881	0.0881	-0.233 to 0.830

^a^ clearance of voriconazole in cancer patients.

^b^ volume of distribution of voriconazole in cancer patients.

^c^ proportional change in clearance with creatinine clearance.

^d^ change in clearance in lymphoma patients.

^e^ change in clearance in Breast cancer patients,

^f^ change in clearance in sarcoma patients,

^g^ change in clearance in myeloma patients,

^h^ change in clearance in glioma patients.

## Discussion

Identification of patient characteristics and the extent of variability among individuals that affect a drug’s pharmacokinetics within a specific population is valuable for dose individualization. Voriconazole, a drug with a narrow therapeutic index, requires careful dosing strategies tailored to each patient to ensure safety and efficacy [20]. The TDM data has been widely used in various population pharmacokinetic studies of voriconazole [15,21,22]. The potential variability in voriconazole plasma concentrations and the corresponding extent were determined using a one-compartment model. The accuracy and stability of this model, which incorporates first-order elimination, were confirmed through evaluation by goodness of fit plots and bootstrap estimates. Majority studies in the literature have confirmed the stability of this model [22–25] but in few studies data were described by two compartment model This shows that one compartment model is better predictor of pharmacokinetics of voriconazole provides sufficient accuracy for dosing adjustments.

The estimated Cl in our study was 6.17 L/h with IIV of 87.2% while the value of clearance reported previously was 11.2 L/h with 21.3% IIV [25]. The clearance of voriconazole in our data set is lower but with high inter individual variability as compared to previously reported. This variation may be due to differences in the disease status in studied population. Another study reported voriconazole clearance as 5.2 L/h with 40% IIV [23]. In our study the value of Vd was 55.9 L which is close to previously reported 68.7 L [25] but differs from another study that reported Vd to be 92 L [23]. This difference is due to differences in study population and concomitant drug administration.

The results showed that primary diagnosis and creatinine clearance were significant covariates affecting clearance of voriconazole. Our findings are different than reported previously in which study population was significant covariate for Vmax [13] and in another study CRCL does not retained in the final model [26]. The other covariates including age, gender, AST/ALT/ALP reflect no significant effects on clearance of voriconazole that provide similar results that are published previously [27] with the exception of one study in which age, and ALP were significant covariates [24]. Weight was significant covariate for voriconazole clearance in studies that utilized two compartment model [28], however, in our study it was non-significant reflecting methodological variation. Many previous studies have evaluated the effect of covariates like age, weight, gender, AST/ALT/ALP but only one study evaluated CRCL in critically ill patients with pulmonary diseases [21]. In cancer population there is no reported study that evaluated type of cancer (primary diagnosis) as a covariate through one compartment Pk model. Up to our knowledge this is first study from Pakistan evaluating the CRCL and primary diagnosis as a factor responsible for variation in clearance of voriconazole in cancer population.

## Conclusion

A population pharmacokinetic model for voriconazole showed that the clearance of voriconazole was significantly affected by CRCL and the primary diagnosis (type of cancer). This model can be applied to customize voriconazole dosing for cancer patients with systemic fungal infections. The findings from this model provide crucial insights into how voriconazole behaves in cancer patients, which can help optimize therapeutic outcomes. By incorporating individual patient characteristics, the model ensures that dosing regimens are more precise and effective.

### Study limitation

The study has few limitations. Firstly, the influence of CYP 2C19 genotype as covariate couldn’t be tested due to non-availability of genotyping facilities in the hospital thus lack of data availability for interpretation. Secondly the effects of concomitant drug administration could not be analyzed due to in complexity in the available data regarding dosing regimens, timing, and specific pharmacokinetic profiles for these drugs. Thirdly, the population included in this study was very heterogeneous in term of age and disease type. More studies are required to observe the interindividual variability by categorizing the diverse age range.

### Future recommendations

Future studies should include CYP 2C19 genotyping as a covariate to better understand its impact on voriconazole pharmacokinetics. Furthermore, designing prospective studies for monitoring the effect of concomitant drug administration would provide more comprehensive insights and improve the interpretability of the findings.

## Supporting information

S1 Data_Voriconazole Dataset used for model development. (CSV)
